# Localized Candidiasis in Kidney Presented as a Mass Mimicking Renal Cell Carcinoma

**DOI:** 10.1155/2012/953590

**Published:** 2012-03-04

**Authors:** Zhao Song, Nicholas Papanicolaou, Stephanie Dean, Zhanyong Bing

**Affiliations:** ^1^Department of Surgery, Jinan Central Hospital, Jinan 250021, China; ^2^Department of Radiology, Hospital of the University of Pennsylvania, 6 Founders, 3400 Spruce Street, Philadelphia, PA 19104, USA; ^3^Department of Pathology and Laboratory Medicine, Hospital of the University of Pennsylvania, 6 Founders, 3400 Spruce Street, Philadelphia, PA 19104, USA

## Abstract

*Candida albicans* is a ubiquitous fungus and infection of urinary tract by *C. albicans* can be originated from blood or retrograde infection. We reported a case of localized candidiasis in the kidney presenting as a mass. The patient was a 61-year-old male with a history of type 2 diabetes mellitus and urinary bladder urothelial carcinoma status post radical cystoprostatectomy with a neobladder three years ago. Pathology at that time also showed a prostatic adenocarcinoma (Gleason score 3 + 4) in addition to the high-grade urothelial carcinoma. Three month ago the patient presented with flank pain, chill, and increased white cell counts. Imaging study showed a large renal mass suspicious for a renal cell carcinoma. Radical nephrectomy was performed and found that there was a large pocket of pus in the retroperitoneum around the right kidney during the surgery. Intraoperative abscess cultures were positive for *C. albicans*. Pathology showed a 13.5 cm necrotic renal mass extending to the perinephric fat. Histologically the tumor showed necrotic granulomatous inflammation. Grocott stain in the surgical specimen was positive for pseudohyphae and yeast forms. The patient was initiated a course of fluconazole postoperatively and was feeling well.

## 1. Introduction

*Candida albicans* is a ubiquitous fungus and can be detected in approximately 32–55% of health individuals [1]. Infection of urinary tract by *C. Candida* can be from blood or retrograde infection [2]. Candidiasis can occasionally present as mass lesions [3, 4]. Localized renal Candida infection is rare. It can be a renal fungal ball [5–8], or rarely can Candida renal infection present as a granulomatous pyelonephritis [9, 10]. In this report, we described a renal candidiasis in a 61-year-old patient presenting as a mass, mimicking renal cell carcinoma.

## 2. Clinical History

**Figure 1:**
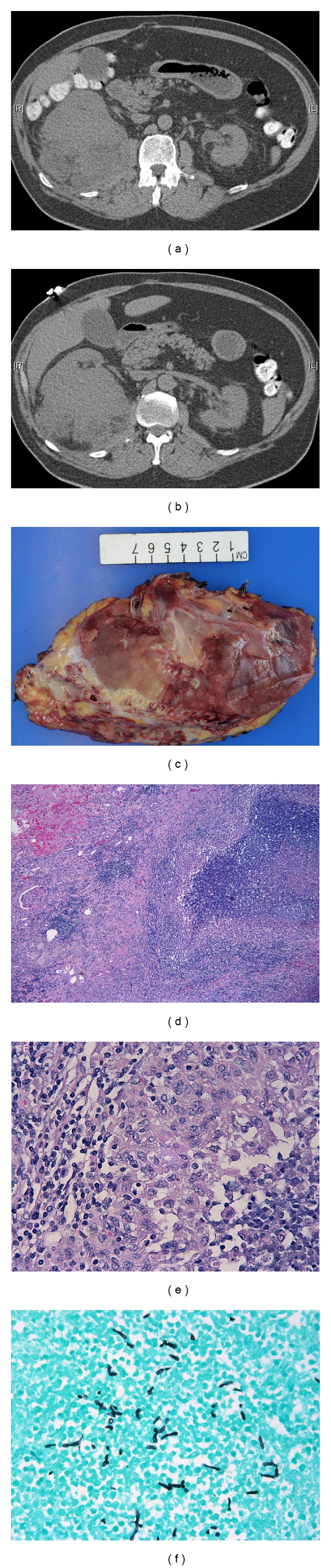
(a) Unenhanced CT of the abdomen displays a large, contour deforming mass arising in the lower pole of the right kidney. The mass contained no visible calcifications, involved the renal capsule, and infiltrated the perinephric fat. (b) More inferiorly, the mass clearly involved the ipsilateral psoas muscle and, after going through the perinephric space and fascia, it extended into the posterior paranephric space. There is no retroperitoneal lymphadenopathy. The right renal vein was of normal caliber on higher slices through the renal hilum. (c) Gross examination. The mass involved renal parenchyma with extrarenal extension. (d) Granulomatous inflammation with abscess involved renal parenchyma, H&E, ×50. (e) Granuloma with adjacent abscess was seen in the renal parenchyma, H&E, ×400. (f) Grocott stain was positive for pseudohyphae and yeast forms, ×400.

The patient was a 61-year-old male with a history of lynch syndrome, dyslipidemia, hypertension, and type II diabetes mellitus and coronary disease and underwent 4-vessel coronary artery bypass graft surgery 17 years ago. He had a smoking history of 1 PPT for 25 years and quitted 18 years ago. Three years ago, he was diagnosed with high-grade urothelial carcinoma in the urinary bladder and underwent cystoprostatectomy with a Studer pouch urinary diversion. Pathologic examination also found an prostatic adenocarcinoma (Gleason score 3 + 4) in addition to the high-grade urothelial carcinoma. Three months ago, the patient presented with right flank pain, chills, and increased white blood cell counts of 23,000. Imaging study showed a complex soft tissue mass of the posterior lower pole of the right kidney and posterior pararenal space. In its entirety, this mass measured 10.7 × 9.6 × 11.9 cm (Figures 1(a) and 1(b)). A portion of this mass was within the kidney and measured approximately 7.2 × 10.2 cm. A portion of this mass which was outside the kidney was heterogeneous and ill defined. There were no calcifications. There was a mildly prominent lymph node posterior to the inferior vena cava, though not demonstrating pathologic enlargement. There was no hydronephrosis. The left kidney was mildly atrophic and contained a 12 mm cyst. Urine culture was positive for yeast. The patient was placed on fluconazole. Upon reviewing his imaging with multiple radiologists including the genitourinary radiologist, it was determined that the mass was suspicious for a renal cell carcinoma. Therefore, decision was made to proceed with right radical nephrectomy. The patient underwent the surgery two months ago and was found to have a large pocket of pus in the retroperitoneum around the right kidney during the surgery. Cultures from the abscess were positive for C. Albicans. Postoperatively the patient was placed on fluconazole, 400 mg, PO after each hemodialysis (HD) for 4 weeks. Patient was put on HD as preoperative renal function scan showed that his right kidney had dominant function, with the left kidney only contributing 18%.The patient had finished the course of fluconazole and he was felling well and denied any fevers, chills, or sweats. He continued to make urine approximately 750 mL daily and had no problems with urination. No hematuria or dysuria was found.

### 2.1. Pathology Findings

The specimen was processed routinely. The specimen consisted of right kidney and perinephric adipose tissue measuring 24.6 × 14 × 6.5 cm and weighed 783.7 grams. On the posterior surface there was a large, necrotic defect mass measuring 13.5 cm in greatest dimension. On cut surface, it showed a large, poorly circumscribed, heterogeneous, and necrotic mass in the lower pole of the kidney measuring 13.0 × 5.0 × 6.0 cm (Figure 1(c)) and extending to the perinephric fat. Histologically the mass was composed of acute and chronic inflammation with abscess formation, fibrosis, and granulomas (Figures 1(d) and 1(e)). Gram, acid fast, and Grocott stains were performed with adequate controls. Grocott stain for fungus was positive for pseudohyphae and yeast forms (Figure 1(f)) compatible with *Candida spp*. Gram and acid fast stains were negative.

## 3. Discussion

Our understanding of renal infection by *C. Albicans* is greatly advanced though the study of animal model. It has been shown that this fungus can be detected in all main organs, especially in the brain or kidneys [11] shortly after inoculation. In the kidneys, yeasts pass through the vascular walls into both of the cortex and medulla, attracting neutrophilic infiltration. Infection in the kidneys, as contrasted to other organs, is not controlled [11, 12]. In the first 12 h, yeast forms elongate and rupture from the interstitium into renal tubules, produce germ tubes and markedly proliferate and elongate. Mycelial casts move down into the medulla and are caught in the loop of Henle, here elongated hyphae can rupture into the interstitium and cause an inflammation reaction mainly composed of mononuclear cells. The hyphae gradually fractured and disappeared. Two weeks after the inoculation, only cellular scars are left in the cortex. If the initial inocula are too much, mice can die due to organ failure and sepsis. The kidneys have diffusely scattered abscess. If the inocula are sublethal, a so-called excretory lesion can be resulted, which mainly confined to the renal pelvis, collecting ducts and ureter [2].

 Candida cause diseases in human when the body defense is compromised such as diabetes mellitus, human immunodeficiency virus infection, cancers, neutropenia, or immunosuppression due to organ transplantation, or when the patients undergo certain procedures such as bladder catheterization or urologic procedure [13]. Patient with type II diabetes mellitus have more infections and course of infections is more complicated. One possible explanation for this is a defect in immune response. It has been shown that a TH2-axis shift, which decreases TH1-dependent immunity. In addition, decrease in cytokine response after stimulations or low-complement factor 4 may contribute for the compromise of humoral innate immunity. It has also been demonstrated that there are decreased functions in chemotaxis and phagocytosis in diabetic PMNs and monocytes [14].

*C. Albicans* infection can present as a mass lesion and have been reported in stomach [15], pancreas [16], liver [17], and hand [18]. Couple of cases of granulomatous pyelonephritis have been reported in kidneys [9, 10]. Mass-forming property may be related to the fungal ability to produce pseudohyphae. *C. glabrata* does not produce true hyphae and cannot produce pseudohyphae except under special cultural conditions. This hyphaeless fungus rarely produces renal mass in the renal pelvis [19].

*C. Albicans* infection in the urinary system can occur through two ways, one is through the blood to spread into the renal parenchyma, and the other is retrograde through the urinary tract [2].

In our case, the mass is composed of granulomatous inflammation, necrosis, and abscess consistent with xanthogranulomatous pyelonephritis (XGP) [20]. No tumor was identified in a well-sampled nephrectomy specimen. XGP is a chronic granulomatous inflammation, most commonly occurred in middle-aged women [9, 20]. The most common microorganisms are *Escherichia coli *and *Proteus mirabilis *[21–24]. Candida can be a cause but is very rare [9, 10]. Computed tomography (CT) is a major diagnostic tool. The findings include hydronephrosis (90.9%), renal stones (72.7%), pyonephrosis (45.5%), intraparenchymatous collection (45.5%), cortical renal atrophy (45.5%), nonfunctioning kidney (36.4%), abscess (36.4%), and perinephric fat accumulation (18.2%) [25]. In our case the imaging findings are not typical. We found a heterogeneous and ill-defined mass involving the kidney with extension into outside of kidney. No real calculus or hydronephrosis was identified albeit the patient underwent cystoprostatectomy with neobladder three years ago.

 All in all, in our case, patient's previous history of bladder cancer status after bladder resection in combination of type 2 diabetic mellitus and smoking may all contribute the infection.

 In summary, *C. Albicans* is a rare cause for xanthogranulomatous pyelonephritis, which can mimic various benign and malignant conditions. Treatments include nephrectomy and antifungal medications.
